# Occupational and domestic physical activity and diabetes risk in adults: Results from a long-term follow-up cohort

**DOI:** 10.3389/fendo.2022.1054046

**Published:** 2022-12-09

**Authors:** Jialu Wang, Liyun He, Na Yang, Ziyi Li, Lingling Xu, Wei Li, Fan Ping, Huabing Zhang, Yuxiu Li

**Affiliations:** Department of Endocrinology, Key Laboratory of Endocrinology of National Health Commission, Peking Union Medical College Hospital, Chinese Academy of Medical Sciences & Peking Union Medical College, Beijing, China

**Keywords:** physical activity, diabetes mellitus, occupational physical activity, domestic physical activity, China Health and Nutrition Survey

## Abstract

**Background:**

Physical activity (PA) has been associated with decreased incidence of diabetes. However, few studies have evaluated the influence of occupational and domestic PA on the risk of diabetes with a long-term follow-up. We aimed to examine the association between occupational and domestic PA and the risk of diabetes in a long-term prospective cohort of Chinese adults.

**Methods:**

A total of 10,343 adults who were followed up in the China Health and Nutrition Survey from 1997 to 2015 were included in our analysis. Occupational and domestical PA were collected with detailed seven-day data and were converted into metabolic equivalents values. Total PA included occupational, domestic, transportation, and leisure time PA. Diabetes cases were identified by self-reported doctor/health professional diagnosis of diabetes, fasting blood glucose ≥7.0 mmol/L, and glycosylated hemoglobin (HbA1c) ≥6.5%. Cox proportional hazards models were used to calculate hazard ratios (HR) and 95% confidence intervals (CI).

**Results:**

During up to 18 years of follow-up (median 10 years), there were 575 diabetes cases documented. Occupational PA accounted for the majority of total PA (68%) in Chinese population, followed by domestic PA (25%). With adjustments for possible covariates, the highest quartiles of total PA (HR, 0.728 [95% CI, 0.570–0.929]) and occupational PA (HR, 0.765 [95% CI, 0.596–0.982]) were significantly associated with a lower risk of diabetes compared with lowest quartiles. The association between domestic PA and the risk of diabetes was insignificant (*P >*0.05).

**Conclusion:**

Higher levels of occupational PA were associated with a decreased risk of diabetes risk in the Chinese population. Domestic PA was not associated with the incidence of diabetes.

## Introduction

The incidence of diabetes mellitus (DM) is increasing and has become a major healthcare burden worldwide, especially in China (1, 2). DM is associated with significant morbidity and mortality (3). Therefore, prevention of DM is an effective way to reduce diabetes related complications and mortality. Physical activity (PA) is a key lifestyle factor in the prevention and management of diabetes. Guidelines for the treatment of the type 2 DM always include recommendations for regular PA (4, 5). Several studies have demonstrated a significant negative association between PA levels and the incidence of DM in adults across different populations (6–8). PA can be divided into different types. According to the World Health Organization (WHO), PA involves work, transportation, leisure, and household chores (9). The patterns of PA have also changed in recent years. For example, a reduction in transportation activity, occupational activity, and domestic activity, but sedentary activity increased (10). The exact relationship between the different PA types and the risk of DM remains unclear.

Exploring the relationship between different types of PA and the risk of DM has important public health implications. A meta-analysis based on prospective studies found that all types of PA help prevent diabetes (6). However, most previous studies on the effect of PA on diabetes risk have focused only on leisure time or total PA (6, 11, 12). Only a few studies have divided total PA into leisure time and occupational PA or combined work-related PA with other types of PA (13–17). However, those previous results on the relationship between occupational PA and the risk of DM have been inconsistent (18–21). Few studies have explored the relationship between domestic PA and the risk of DM (21, 22). Thus, this information has rarely been studied, particularly in the Chinese population.

Therefore, this study aimed to explore the association between occupational and domestic PA and the risk of diabetes in the Chinese adult population using the China Health and Nutrition Survey (CHNS) database and to clarify which type of PA plays a major role. To our knowledge, this is the first large-scale, nationally representative, long-term follow-up to evaluate the relationship between occupational and domestic PA and the risk of DM risk in Chinese adults.

## Materials and methods

### Study population

The CHNS is an ongoing open nationwide cohort study in China was designed to investigate the impact of social and economic transformation on the health and nutritional status of the Chinese population (23). The study was conducted with international collaboration between the University of North Carolina at Chapel Hill and the National Institute of Nutrition and Food Safety of the Chinese Center for Disease Control and Prevention, with CHNS data provided on the website (https://www.cpc.unc.edu/projects/china). The survey used a multistage randomized cluster sampling method, with samples drawn from 15 provinces (Beijing, Chongqing, Guangxi, Guizhou, Heilongjiang, Henan, Hubei, Hunan, Jiangsu, Liaoning, Shaanxi, Shandong, Shanghai, Yunnan, and Zhejiang) in mainland China, covering most of the northern and southern regions. The CHNS conducted 10 rounds of surveys between 1989 and 2015, with 7 days of examination and data collection by researchers in the fields of nutrition, public health, economics, and sociology (24). Further details and information about the CHNS can be found elsewhere (25).

This present study enrolled 32,752 participants without diabetes at baseline in the CHNS from 1997 to 2015 because information about occupational PA was unavailable before 1997. Furthermore, 22,409 participants were excluded based on the following criteria: age <18 years, missing occupation and domestic PA data, missing diabetes information, who were pregnant or breastfeeding, physically disabled participants, without complete follow-up data, and those with a history of myocardial infarction (MI), stroke, or any type of tumor at baseline.

All research procedures were conducted in accordance with the tenets of the Declaration of Helsinki (as revised in 2013) and were approved by the institutional review boards of the University of North Carolina at Chapel Hill, the National Institute for Nutrition and Health, and the Chinese Center for Disease Control and Prevention. Written informed consent was obtained from each participant.

### Diabetes definition

Diabetes status was collected using a staff-administered questionnaire at each follow-up since 1997 and fasting blood samples in 2009. A total of 9,549 CHNS participants’ blood samples were collected. Diabetes cases were identified as those who self-reported a clinical diagnosis of diabetes, or receiving diabetes treatment, or with FBG ≥7.0 mmol/L (126 mg/dL), or with HbA1c ≥6.5%. The treatments for diabetes included in the questionnaires were special diet, weight control, oral medication, insulin injection, traditional Chinese medicine, and home remedies. Blood samples were collected from all participants after 12–14 hours of fasting and were stored in test tubes. All blood samples were analyzed at the central laboratory of the China-Japan Friendship Hospital. Fasting blood glucose was measured with glucose oxidase-peroxidase (GOD-PAP) using a kit manufactured by Landau, UK (26). Further details on the data are available at https://www.cpc.unc.edu/projects/china.

### Physical activity assessment

A staff-administered questionnaire collected the self-reported PA of participants, which gathered detailed PA information in four categories: domestic, occupation, transportation, and leisure activity. Participants answered questions related to the average weekly or daily frequency and duration of each type of PA in the previous year. Metabolic equivalent of task (MET) was defined as the ratio of the working metabolic rate to the standard resting (basal) metabolic rate. The time spent per week for different PAs was multiplied by a specific MET value based on the Compendium of Physical Activities to calculate the average MET hours/week to evaluate the intensity and duration of the activity (27). Occupational activities were categorized into light- (sedentary or occasional standing work), moderate- (such as drivers, electricians), and heavy-intensity (such as farmers, athletes, dancers, steel workers, lumberjacks, construction workers) based on specific self-reported work and assigned values of 2.0, 4.0, and 6.0, respectively. The domestic activity was measured based on the following five activities: buying food, preparing food or cooking, doing laundry, cleaning house, and childcare. Transportation activities were measured based on four commuting types (walking, biking, bus, car) to and from work or school. The evaluation of leisure time activities was based on martial arts, gymnastics or dancing, jogging or swimming, playing ball sports (soccer, basketball, tennis, badminton, volleyball, or ping-pong), and other activities (28). Total PA included occupational, domestic, transportation, and leisure time PA. In our study, three different domains of PA were employed: occupational, domestic, and total PA levels.

### Data collection of covariates

Anthropometrics were measured by trained investigators following the anthropometric standards recommended by the WHO (29). Three measurements were taken for each participant and analyzed using the mean measurements. Participants wore light clothing, and their weight was measured using a calibrated beam scale with a weight measurement accurate to 0.1 kg. The participants’ height without footwear or barefooted was measured using a portable stadiometer, accurate to 0.1 cm. Body mass index (BMI) was calculated as weight (kg) divided by height (meters) squared. Blood pressure was measured three times after resting in a seated position with an interval of 10 minutes and the average of the three measurements was used to calculate systolic and diastolic blood pressure.

The standardized questionnaire was used by the investigators to collect demographic characteristics, health history, and health-related behaviors, including age, sex, marital status, smoking status (current/ever smoking or not), alcohol consumption (current/ever drinking or not), and educational attainment level (low: lower middle school or below; medium: higher middle school or vocational/technical school; high: college/university or higher). Dietary information was collected through a 3-day dietary review using food weighing methods to assess total energy intake.

### Statistical analysis

Continuous variables are expressed as mean ± standard deviation (M ± SD), and categorical data are presented as percentages or frequencies. Participants were divided into four groups according to quartiles of PA, and the lowest quartile was used as a reference. ANOVA tests were used to compare between-group differences for continuous data, whereas chi-squared tests were used to compare between-group differences for categorical data. Kaplan–Meier survival curves were used to assess the relationship between different types of PA and diabetes, and log-rank tests were used to examine significant differences among quartiles. Cox proportional hazards models were used to assess the association between PA and DM, and hazard ratios (HR) and 95% confidence intervals (CI) were calculated. Possible confounding factors were adjusted for in the regression models. Model 1 was adjusted for age and sex; Model 2 was adjusted for the variables in Model 1 plus marital status, education, household per capita income level, and urbanization index; Model 3 was adjusted for the variables in Model 2 plus smoking status, alcohol consumption, total energy intake, and blood pressure; and Model 4 was adjusted for the variables in Model 3 plus BMI. To explore the effect modifications, subgroup analyses were performed according to age (by the median age: <42 years or ≥42 years), sex, BMI (<20, 20–24, >24), smoking status, and alcohol consumption. All statistical analyses were performed using SAS (version 9.4; SAS Institute, Cary, North, USA) and R version 4.0.2. A two-sided test of P<0.05 indicates a statistically significant difference.

## Results

### Baseline characteristics of the participants

**Figure 1:**
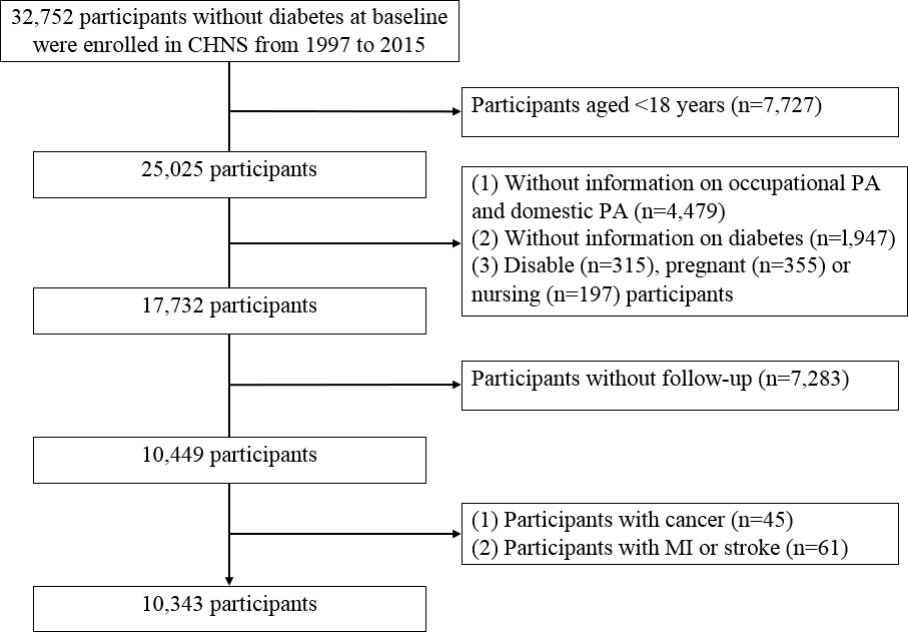
Study population flowchart. Notion: CHNS, China Health and Nutrition Survey; PA, physical activity; MI, myocardial infarction.

A total of 32, 752 participants were eligible for enrollment in the study, and 10,343 participants were finally included based on the exclusion criteria (**Figure 1**). In the overall study population, the mean age was 41.1(12.9) years, 43.67% were men, and the mean BMI was 22.7 kg/m^2^(3.3) at baseline. During the 18 years follow-up (median 10.3 years), 575 incident cases of DM were documented, and the mean age of diagnosed DM was 56.1 years. The mean total PA, occupational PA, and domestic PA were 207.3 (127.7) MET·h/week, 149.1 (108.0) MET·h/week, and 44.6 (57.0) MET·h/week, respectively. The baseline characteristics of the participants are presented in **Table 1** according to quartiles of total PA, occupational PA, and domestic PA. **Table S1** describes the baseline profile of the study population according to sex (30).

**Table 1:** Baseline characteristic of the study population.

			Total PA		Occupational PA					Domestic PA					
	Q1(<115 MET·h/week)	Q2(115-182 MET·h/week)	Q3(182-276 MET·h/week)	Q4(276-1671 MET·h/week)	P value	Q1(<78 MET·h/week)	Q2(78-120 MET·h/week)	Q3(120-216 MET·h/week)	Q4(216-1208 MET·h/week)	P value	Q1(<13 MET·h/week)	Q2(13-31 MET·h/week)	Q3(31-53 MET·h/week)	Q4(53-536 MET·h/week)	P value
N	2584	2586	2586	2587	NA	2584	2586	2586	2587	NA	2584	2586	2586	2587	NA
Age (y)	44.0 ± 14.8	41.1 ± 12.5	40.0 ± 12.1	39.5 ± 11.3	<.0001	44.3 ± 14.5	40.1 ± 12.4	40.3 ± 12.3	39.3 ± 11.6	<.0001	40.0 ± 13.7	41.9 ± 13.5	47.7 ± 12.2	40.0 ± 11.7	<.0001
Male (n, %)	1227 (47.5)	1068 (41.3)	1102 (42.6)	1120 (43.3)	<.0001	999 (38.7)	1076 (41.6)	1149 (44.4)	1293 (50.0)	<.0001	2108 (81.6)	1314 (50.8)	630 (24.4)	465 (18.0)	<.0001
Married (n, %)	2116 (81.9)	2265 (87.6)	2292 (88.6)	2307 (89.2)	<.0001	2194 (84.9)	2236 (86.5)	2259 (87.4)	2291 (88.6)	0.0010	2056 (79.6)	2211 (85.5)	2313 (89.4)	2400 (92.8)	<.0001
Education					<.0001					<.0001					0.0009
Low (n, %)	1639 (63.4)	1605 (62.1)	2037 (78.8)	2254 (87.1)		1774 (68.7)	1313 (50.8)	2110 (81.6)	2338 (90.4)		1844 (71.4)	1819 (70.3)	1949 (75.4)	1923 (74.3)	
Medium (n, %)	637 (24.7)	691 (26.7)	448 (17.3)	298 (11.5)		583 (22.6)	830 (32.1)	423 (16.4)	238 (9.2)		551 (21.3)	561 (21.7)	474 (18.3)	488 (18.9)	
High (n, %)	308 (11.9)	290 (11.2)	101 (3.9)	35 (1.4)		227 (8.8)	443 (17.1)	53 (2.1)	11 (0.4)		189 (7.3)	206 (8.0)	163 (6.3)	176 (6.8)	
Urban Index	65.0 ± 20.0	66.0 ± 19.8	56.3 ± 20.0	48.6 ± 16.4	<.0001	63.6 ± 19.5	69.6 ± 19.3	55.5 ± 19.3	47.0 ± 15.1	<.0001	57.8 ± 19.7	60.6 ± 20.3	58.3 ± 20.8	59.1 ± 20.3	<.0001
Total Energy intake (kcal/d)	2189.5 ± 1520.4	2129.0 ± 663.4	2252.0 ± 635.9	2350.2 ± 728.6	<.0001	2141.5 ± 980.3	2140.6 ± 1322.6	2246.5 ± 644.5	2391.8 ± 738.4	<.0001	2409.7 ± 1014.0	2238.1 ± 1310.6	2172.4 ± 720.4	2100.6 ± 629.4	<.0001
Ever/current smoker (n, %)	798 (30.9)	696 (26.9)	789 (30.5)	803 (31.0)	0.0026	669 (25.9)	681 (26.3)	801 (31.0)	935 (36.1)	<.0001	1356 (52.5)	860 (33.3)	501 (19.4)	369 (14.3)	<.0001
Alcohol consumer (n, %)	945 (36.6)	904 (35.0)	911 (35.2)	947 (36.6)	0.4649	824 (31.9)	929 (25.9)	931 (36.0)	1023 (39.5)	<.0001	1471 (56.9)	1046 (40.5)	640 (24.8)	550 (21.3)	<.0001
BMI (kg/m2)	23.0 ± 3.6	23.0 ± 3.3	22.7 ± 3.2	22.4 ± 3.0	<.0001	22.9 ± 3.5	23.0 ± 3.3	22.6 ± 3.2	22.3 ± 3.1	<.0001	22.6 ± 3.4	22.6 ± 3.3	22.9 ± 3.2	22.9 ± 3.2	0.0001
SBP (mm Hg)	120.0 ± 17.1	117.8 ± 16.3	116.4 ± 16.0	117.2 ± 16.0	<.0001	119.8 ± 17.5	117.5 ± 16.0	116.7 ± 16.1	117.5 ± 15.7	<.0001	118.8 ± 15.1	118.2 ± 16.2	118.3 ± 17.5	116.3 ± 16.7	<.0001
DBP (mm Hg)	78.1 ± 11.1	77.5 ± 11.0	76.4 ± 10.7	76.9 ± 11.0	<.0001	77.8 ± 11.1	77.4 ± 10.9	76.6 ± 10.8	77.0 ± 11.0	0.0002	78.2 ± 10.8	77.3 ± 10.9	77.1 ± 11.1	76.3 ± 10.8	<.0001

Data given as mean ± SD, n (%), or median (interquartile range).

NA, not applicable; BMI, body mass index; SBP, systolic blood pressure; DBP, diastolic blood pressure; PA, physical activity; WC, waist circumference.

As total and occupational PA increased, BMI, SBP, and DBP decreased, total energy intake increased, and the proportion of young people, married, with low education level, and smoking also showed a tendency to increase. However, with an increase in domestic PA, the proportion of men, smokers, and alcohol drinkers declined significantly, and energy intake decreased. Men were more likely to have higher occupational PA, energy intake, BMI, and BP, whereas women were more likely to have higher domestic PA, almost twice as much as men were. **Table S2** summarizes the different domains of PA in the study population at baseline (30). Among the Chinese population, occupational PA accounted for the majority of total PA (68%), followed by domestic PA (25%), while transportation and leisure PA accounted for only a small percentage (7%).

### Association of total, occupational, domestic PA with DM

**Figure 2:**
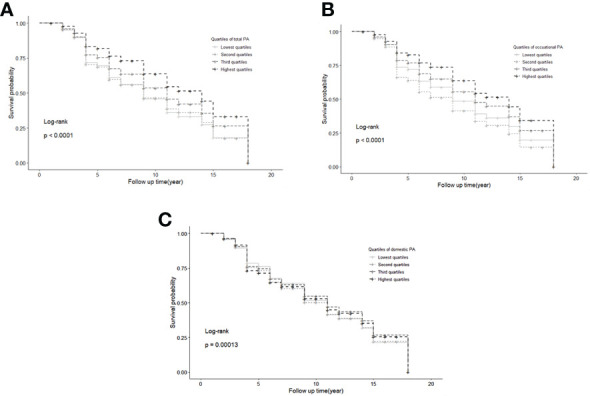
Kaplan-Meier estimates of diabetes incidence by quartiles of total **(A)**, occupational **(B)**, and domestic physical activity **(C)**. Notion: PA, physical activity.

PA was divided into quartiles, with the lowest quartile considered as the reference group. Kaplan–Meier estimates of DM incidence by quartiles showed that the highest quartile of total PA and occupational PA had the lowest risk of DM (**Figure 2**), with significant differences in survival time determined by log-rank statistics (P <0.0001). The log-rank statistics for domestic PA also showed differences in survival time among the four groups (P =0.00013).

Multivariate-adjusted Cox proportional hazard models were used to assess the association between PA and DM. In Models 1, 2, and 3, the highest quartiles of total PA and occupational PA were inversely associated with the risk of diabetes. Further adjustment for BMI in Model 4 attenuated this association, but the highest quartiles of total PA HR = 0.728 [95% CI, 0.571–0.930] and occupational PA HR = 0.765 [95% CI, 0.596–0.982] were still associated with a reduced risk of DM. However, no significant association was observed between domestic PA and the risk of DM after adjusting for various factors (**Table 2**).

**Table 2:** HRs (95% CI) of diabetes according to the quartiles of total PA, occupational PA, and domestic PA.

	No. of participants/DM	Model 1	Model 2	Model 3	Model 4
Total PA					
Q1 (lowest)	10343/172	Reference	Reference	Reference	Reference
Q2	10343/138	0.862 (0.698, 1.079)	0.835 (0.667, 1.046)	0.831 (0.663, 1.041)	0.849 (0.678, 1.064)
Q3	10343/144	0.785 (0.625, 0.986)	0.795 (0.630, 1.003)	0.825 (0.654, 1.042)	0.876 (0.693, 1.105)
Q4 (highest)	10343/121	0.654 (0.517, 0.827)	0.670 (0.525, 0.855)	0.670(0.524, 0.856)	0.728 (0.570, 0.929)
P value for trend		0.0043	0.0015	0.0022	0.0179
Occupational PA					
Q1 (lowest)	10343/172	Reference	Reference	Reference	Reference
Q2	10343/138	1.024 (0.817, 1.284)	1.000 (0.796, 1.256)	1.006 (0.801, 1.264)	1.012 (0.805, 1.272)
Q3	10343/144	0.865 (0.691, 1.083)	0.877 (0.697, 1.103)	0.910 (0.723, 1.144)	0.957 (0.761, 1.204)
Q4 (highest)	10343/121	0.657 (0.581, 0.834)	0.684 (0.533, 0.877)	0.699 (0.544, 0. 898)	0.765 (0.596, 0.982)
P value for trend		0.0012	0.0029	0.0066	0.0492
Domestic PA					
Q1 (lowest)	10343/172	Reference	Reference	Reference	Reference
Q2	10343/138	0.967 (0.761, 1.230)	0.931 (0.732, 1.185)	0.939 (0.738, 1.196)	0.968 (0.760, 1.232)
Q3	10343/144	0.980 (0.758, 1.268)	0.941 (0.727,1.217)	0.907 (0.701, 1.175)	0.906 (0.699, 1.176)
Q4 (highest)	10343/121	0.994 (0.761, 1.299)	0.942 (0.721, 1.231)	0.913 (0.698, 1.195)	0.879 (0.672, 1.150)
P value for trend		0.9926	0.7213	0.5570	0.3067

Model 1 adjusted for age and gender (male or female).

Model 2 adjusted for age and gender (male or female), marriage status (married or not), educational attainment levels (low, medium, or high), household income per capita levels (low, medium, or high), and urbanization index.

Model 3 was adjusted for age and gender (male or female), marriage status (married or not), educational attainment levels (low, medium, or high), household income per capita levels (low, medium, or high), urbanization index, smoking status (ever/current or never smoker), alcohol consumption (yes or no), total energy intake, SBP, and DBP.

Model 4 was adjusted for age and gender (male or female), marriage status (married or not), educational attainment levels (low, medium, or high), household income per capita levels (low, medium, or high), and urbanization index, smoking status (ever/current or never smoker), alcohol consumption (yes or no), total energy intake, SBP, DBP and BMI.

HR, hazard ratio; DM, diabetes mellitus; PA, physical activity; SBP, systolic blood pressure; DBP, diastolic blood pressure; BMI, body mass index.

### Subgroup analyses stratified by age, sex, BMI, smoking status and alcohol assumptions

**Figure 3:**
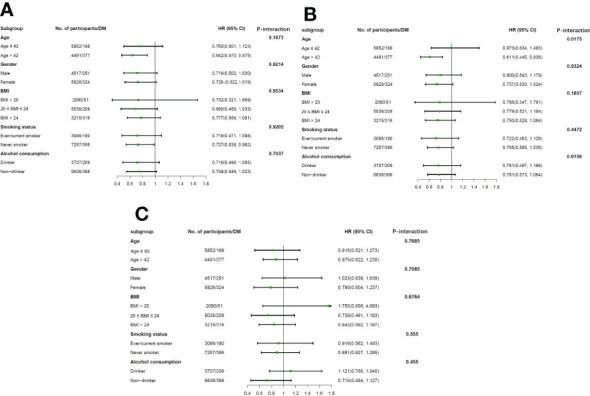
Association of total **(A)**, occupational **(B)**, and domestic physical activity **(C)** with diabetes incidence stratified by age, sex, BMI, smoking status, and alcohol consumption. Notion: All models were adjusted for age and gender (male or female), marriage status (married or not), educational attainment levels (low, medium, or high), household income per capita levels (low, medium, or high), and urbanization index, smoking status (ever/current or never smoker), alcohol consumption (yes or no), total energy intake, SBP, DBP and BMI. The results were the HR (95% CI) of diabetes incidence according to the quartiles of total PA, occupational PA, and domestic PA. HR, hazard ratio; DM, diabetes mellitus; PA, physical activity.

Subgroup analyses were conducted to clarify whether the relationship between total, occupational, and domestic PA and the risk of DM was influenced by other potential factors (**Table 3**), which are also presented in the **Figure 3**. The model was adjusted for age, sex, marital status, education, household per capita income level, urbanization index, smoking status, alcohol consumption, total energy intake, blood pressure, and BMI. Stronger associations of total PA (HR = 0.642 [95% CI, 0.470–0.875]) and occupational PA (HR=0.611 [95% CI, 0.445–0.838]) with the risk of DM were observed in participants aged >42 years than in younger adults (aged ≤42 years). Significant subgroup differences in age were found for occupational PA (P-interaction = 0.0175), but not for total PA (P-interaction >0.05). We further divided the study population into three groups (<40 years, 40–60 years, and >60 years) and observed a decreasing trend in the risk of DM among these groups, although the group differences were insignificant (**Table S3**) (30). No significant modified effects of sex, BMI, smoking status, and alcohol consumption were observed for the associations between total, occupational, and domestic PA and the risk of DM (P-interaction >0.05).

**Table 3:** HRs (95% CI) of the risk of DM according to quartiles of total PA, occupational PA, and domestic PA stratified by age, sex, BMI, smoking status, and alcohol consumption.

	No. of participants/DM	total PA		Occupational PA		Domestic PA	
		HR (95% CI)	P-interaction	HR (95% CI)	P-interaction	HR (95% CI)	P-interaction
Age			0.1673		0.0175		0.7685
Age ≤ 42	5654/198	0.750(0.501, 1.123)		0.973(0.634, 1.493)		0.815(0.521, 1.273)	
Age > 42	4114/377	0.642(0.470, 0.875)		0.611(0.445, 0.838)		0.875(0.622, 1.230)	
Gender			0.9214		0.9324		0.7085
Male	4517/251	0.719(0.502, 1.030)		0.800(0.543, 1.179)		1.023(0.639, 1.638)	
Female	5826/324	0.729 (0.522,1.019)		0.737(0.530, 1.024)		0.790(0.504, 1.237)	
BMI			0.9534		0.1807		0.6764
BMI < 20	2090/51	0.732(0.321, 1.669)		0.786(0.347, 1.781)		1.755(0.656, 4.693)	
20 ≤ BMI ≤ 24	5038/208	0.689(0.459, 1.033)		0.779(0.521, 1.164)		0.739(0.461, 1.183)	
BMI > 24	3215/316	0.777(0.559, 1.081)		0.750(0.528, 1.064)		0.842(0.592, 1.197)	
Smoking status			0.9205		0.4472		0.5550
Ever/current smoker	3086/180	0.719(0.471, 1.098)		0.722(0.463, 1.126)		0.916(0.562, 1.493)	
Never smoker	7257/395	0.727(0.538, 0.982)		0.765(0.565, 1.036)		0.891(0.627, 1.265)	
Alcohol consumption			0.7037		0.6156		0.4550
Drinker	3707/209	0.719(0.486, 1.065)		0.761(0.497, 1.166)		1.121(0.766, 1.640)	
Non-drinker	6636/366	0.748(0.546, 1.023)		0.781(0.573, 1.064)		0.715(0.454, 1.127)	

Results from model, which adjusted for age, gender (male or female), marriage status (married or not), educational attainment levels (low, medium, or high), household income per capita levels (low, medium, or high), urbanization index, smoking status (ever/current or never smoker), alcohol consumption (yes or no), total energy intake, SBP, DBP, and BMI.

HR, hazard ratio; CI, confidence interval; DM, diabetes mellitus; PA, physical activity; SBP, systolic blood pressure; DBP, diastolic blood pressure; BMI, body mass index.

## Discussion

This large prospective study observed significant inverse associations between total and occupational PA and the risk of DM in the Chinese population. The protective effects of PA may mainly be derived from occupational PA. Age-specific differences were observed in the negative association between occupational PA and DM, with stronger correlations in middle-aged and older adults than in younger adults. However, no significant association was observed between domestic PA and the risks of DM.

Previous studies conducted in different populations yielded findings consistent with this study that higher PA is associated with a decreased risk of DM (6–8). Some studies have demonstrated that PA can significantly decrease HbA1c and insulin levels (31–33), which may partly explain the inverse correlation between total PA and the risk of DM.

Similar to total PA, occupational PA was inversely associated with the risk of DM in current study. Several studies using data from the NHANS and the Malmö Diet and Cancer Study consistently showed that occupational PA was negatively associated with DM (16, 21). Moreover, a previous study that used the CHNS data found an inverse association between occupational PA and insulin resistance in non-diabetic population, which might explain the relationship between occupational PA and DM and support our findings (34). However, some studies have not observed an association between occupational PA and the risk of DM (13, 14, 18, 19). A study in the Mexican population selected only low-income groups; thus, the inconsistent results might be due to differences in the study population (19). In a study conducted in Canada, occupational PA levels of the study population were derived from a job exposure matrix of occupation titles, whereas our study population was based on the MET to quantify the occupational PA intensity. The different methods of obtaining PA levels may have contributed to the inconsistent findings (13). In addition, previous studies in Asian populations have found no relationship between occupational PA and the risk of DM, which may be due to different PA patterns in different national populations (14, 18). One study mentioned that transportation PA accounted for the majority of total PA in the Korean population, followed by leisure time PA (14), while occupational PA accounted for the highest proportion and leisure time PA for the lowest proportion of total PA in our study. Our findings suggested that occupational PA accounts for most of the total PA in the Chinese population and that there is a consistent negative relationship between total PA and occupational PA and the risk of DM (10). Therefore, occupational PA may play a protective role in DM prevention, and the risk of DM may be elevated in people with low occupational activities.

To date, relatively few studies have evaluated the relationship between domestic PA and the risk of DM. A previous study in a Korean population combined work and housework PA (DWPA) showed that moderate- or high-intensity DWPA increased the DM risk, but the combination of different types of PA may have biased the results of study (22). A previous study using data from the Malmö diet and Cancer study showed that domestic PA was positively associated with the risk of DM (21), whereas no significant associations were found between domestic PA and the risk of DM in the current study. Undoubtedly, it is important to explore the relationship between domestic PA and the risk of DM. Our findings showed that domestic PA accounted for the second largest proportion of total PA and was one of the main sources of energy expenditure for women (30). Another study of a Chinese population also found that a large percentage of older adults were highly involved in domestic-related PA (35). Moreover, domestic PA typically exercises smaller upper body muscles and is characterized by a long duration and highly repetitive rhythm, which differs from leisure time PA (36). Hence, domestic PA may have different effects on the risks of DM from other domains of PA. The association between domestic PA and DM is not yet conclusive and more studies are required in the future.

In addition, our study found that the inverse association between total and occupational PA and the risk of DM was more pronounced in the middle-aged and older populations over 42 years of age. However, the prevalence of DM increased more sharply after middle-age (37). We further divided population into three groups and observed a declining trend in the risk of DM among the groups, with a significant inverse association between total and occupational PA and the risk of DM in the >60 years group. Therefore, the older adults should be more concerned about the health effects of occupational PA. No significant sex disparities were found, but there was a consistent relationship between total and occupational PA and the risk of DM in Chinese adults. We did not observe statistically significant differences among the different BMI subgroups, although the association between PA and the risk of DM was weakened after the model further adjust for BMI.

Several possible mechanisms may explain the relationship between PA and the risk of DM. PA could increase energy expenditure to prevent obesity, a major risk factor closely associated with diabetes (38, 39). Moreover, PA increased insulin sensitivity, improved lipid metabolism, and regulated hepatic glucose output in both normal and insulin-resistant individuals (33, 34). Furthermore, acute PA could increase glucose uptake by skeletal muscle cells through the transfer of glucose transporter 4 (GLUT4) from intercellular sites to the plasma membrane. Long-term PA is associated with alterations in the skeletal muscle, including increases in GLUT4 protein levels and mitochondrial enzyme content, as well as changes in the types of muscle fibers that facilitate glucose transport (40–42).

The present study has several strengths. First, our study systematically assessed the independent effects of occupational and domestic PA on the risk of DM, rather than focusing solely on total or leisure PA. Second, this is a prospective, large-scale study with a long follow-up period, and included data from nationally representative Chinese populations rather than restricted populations, such as the population limited to the older adults or those with diabetes, which improved the generalizability of our findings to the entire Chinese population. Third, METs were calculated to quantify PA levels in our study, which is more accurate. Nevertheless, this study has some limitations. First, PA information was obtained through a questionnaire, which may have led to recall bias. Second, the diagnosis of diabetes was mainly based on self-reports and blood samples were collected only in 2009. This may have led to an inaccurate incidence rate of DM and biased the Cox model results. Finally, although we adjusted covariates in the regression models, residual confounding from unmeasured or unknown factors remains possible.

In conclusion, to the best of our knowledge, this is the first large-scale, nationally representative study with a long-term follow-up to systematically assess the independent association between occupational PA and domestic PA and the risk of DM in Chinese adults. Our findings showed that occupational PA was negatively associated with the risk of DM, whereas domestic PA was not associated with the risk of DM. Therefore, occupational PA may require more attention. Diabetes could be prevented by carrying out more other types of activities to increase the total amount of PA for people with low level of occupational activities. Individualized diabetes prevention and management strategies should be developed for different occupational groups, especially middle-aged and older people.

## Data availability statement

The raw data supporting the conclusions of this article will be made available by the authors, without undue reservation.

## Ethics statement

The studies involving human participants were reviewed and approved by the institutional review boards of the University of North Carolina at Chapel Hill, the National Institute for Nutrition and Health, and the Chinese Center for Disease Control and Prevention. The patients/participants provided their written informed consent to participate in this study.

## Author contributions

The conception and design of the study: YL, HZ, LH, and JW. Acquisition of the data: JW, LH, ZL, and HZ. Analysis and interpretation of the data: JW, LH, HZ, and WL. Draft of the article: JW, LH and HZ. Critical revision for important intellectual content: JW, LH, NY, LX, FP, WL, HZ, and YL. Final approval of the version to be published: All authors. Funding acquisition: HZ and YL. Supervision: HZ and YL. Agreement to be accountable for all aspects of the work: All authors. All authors have approved the final article.
