# Supplementary material for: Occupational and domestic physical activity and diabetes risk in adults: Results from a long-term follow-up cohort
Source: Front Endocrinol (Lausanne). 2022 Dec 9;13:1054046. doi: 10.3389/fendo.2022.1054046 (PMC9780271; doi:10.3389/fendo.2022.1054046)
Supplement: Supplementary file 1 [file Table_1.docx]

Table S1. Baseline characteristic of the study population according to sex.

|  | Men  (n = 4517) | Women  (n = 5826) | P value |
| --- | --- | --- | --- |
| Age (y) | 41.7±13.0 | 40.7±12.7 | <0.0001 |
| Married (n, %) | 3827 (84.74) | 5153 (88.45) | <0.0001 |
| Education |  |  | <0.0001 |
| Low (n, %) | 3072 (68.0) | 4463 (76.6) |  |
| Medium (n, %) | 1076 (22.8) | 998 (17.1) |  |
| High (n, %) | 369 (8.2) | 365 (6.3) |  |
| Urban Index | 60.0±20.2 | 58.1±20.3 |  |
| Total Energy intake (kcal/d) | 2435.3±750.2 | 2071.2±1073.9 | <0.0001 |
| Carbohydrate (g/d) | 350.1±124.1 | 307.1±112.7 | <0.0001 |
| Fat intake (g/d) | 77.3±53.5 | 65.6±106.9 | <0.0001 |
| Protein intake  (g/d) | 74.3±25.7 | 62.7±20.6 | <0.0001 |
| Ever/current smoker (n, %) | 2883 (63.8) | 203 (3.5) | <0.0001 |
| Alcohol consumers (n, %) | 3070 (68.0) | 637 (10.9) | <0.0001 |
| Total PA (MET·h/week) | 205.5±130.6 | 208.7±125.4 | 0.2121 |
| Time of total PA (hours/week) | 56.8±34.2 | 64.9±34.3 | <0.0001 |
| Occupational PA (MET·h/week) | 160.5±110.5 | 140.3±105.2 | <0.0001 |
| Time of occupational PA (hours/week) | 40.3±21.2 | 37.4±21.6 | <0.0001 |
| Domestic PA (MET·h/week) | 29.6±53.0 | 56.3±57.2 | <0.0001 |
| Time of domestic PA (hours/week) | 12.6±22.7 | 24.0±23.6 | <0.0001 |
| BMI (kg/m2) | 22.8±3.3 | 22.6±3.2 | 0.0021 |
| WC (cm) | 81.4±10.1 | 76.9±9.3 | <0.0001 |
| SBP (mm Hg) | 120.7±15.3 | 115.7±16.9 | <0.0001 |
| DBP (mmHg) | 79.3±10.9 | 75.6±10.7 | <0.0001 |

Data given as mean ± SD, n (%), or median (interquartile range).

Abbreviations: BMI, body mass index; SBP, systolic blood pressure; DBP, diastolic blood pressure; PA, physical activity; WC, waist circumference.

Table S2. Summary of different domains of physical activity at baseline.

|  | Total  (n = 10343) | | Men  (n = 4517) | | Women  (n = 5826) | |
| --- | --- | --- | --- | --- | --- | --- |
|  | Mean | Median | Mean | Median | Mean | Median |
| Total PA | 207.3 (127.7) | 182.5 (161.2) | 205.5 (130.6) | 179.3 (165.0) | 208.7 (125.4) | 184.0 (158.1) |
| Occupation PA | 149.1 (108.0) | 120.0 (138.0) | 160.5 (110.5) | 140.0 (156.0) | 140.3 (105.2) | 112.0 (130.0) |
| Domestic PA | 44.6 (57.0) | 30.8 (39.6) | 29.6 (53.0) | 15.5 (24.2) | 56.3 (57.2) | 42.8 (36.1) |
| Transportation PA | 9.5 (43.1) | 3.8 (0) | 9.6 (41.9) | 3.3 (10.0) | 9.4 (44.1) | 3.8 (10.0) |
| Leisure time PA | 4.1 (17.0) | 0 (0) | 5.9 (21.4) | 0 (0) | 2.7 (12.3) | 0 (0) |

Data given as mean (SD), or median (interquartile range). Abbreviation: PA, physical activity.

Table S3. HRs (95% CI) of the risk of DM according to quartiles of total, occupational, and domestic physical activity stratified by age.

|  | No. of participants/DM | total PA | | Occupational PA | | Domestic PA | |
| --- | --- | --- | --- | --- | --- | --- | --- |
|  |  | HR (95% CI) | P-interaction | HR (95% CI) | P-interaction | HR (95% CI) | P-interaction |
| Age |  |  | 0.9137 |  | 0.2419 |  | 0.7665 |
| < 40 | 4954/132 | 0.714(0.431, 1.183) |  | 0.961(0.544, 1.697) |  | 0.707(0.416, 1.999) |  |
| 40-60 | 4435/361 | 0.741(0.546, 1.005) |  | 0.738(0.545, 1.000) |  | 0.924(0.650, 1.315) |  |
| > 60 | 954/82 | 0.369(0.153, 0.889) |  | 0.339(0.131, 0.878) |  | 0.698(0.321, 1.519) |  |

Results from model, which adjusted for age, gender (male or female), marriage status (married or not), educational attainment levels (low, medium, or high), household income per capita levels (low, medium, or high), urbanization index, smoking status (ever/current or never smoker), alcohol consumption (yes or no), total energy intake, SBP, DBP, and BMI.

Abbreviations: HR, hazard ratio; CI, confidence interval; DM, diabetes mellitus; PA, physical activity; BMI, body mass index; SBP, systolic blood pressure; DBP, diastolic blood pressure.
